# The Effect of Head‐Forward Posture on Lower Neck Loads and Dislocation Risk in Head‐First Impact: A Computational and Experimental Investigation

**DOI:** 10.1002/jsp2.70219

**Published:** 2026-09-07

**Authors:** Mario Mongiardini, Duane Cronin, Ryan D. Quarrington

**Affiliations:** ^1^ Centre for Automotive Safety Research Adelaide University Adelaide South Australia Australia; ^2^ Department of Mechanical and Mechatronics Engineering University of Waterloo Waterloo Ontario Canada; ^3^ SpineLabs, College of Health Adelaide University Adelaide South Australia Australia

**Keywords:** cervical facet dislocation, finite element analysis, head‐first impact, head‐forward posture, human body model

## Abstract

**Background:**

Subaxial cervical facet dislocation (CFD) is a severe neck injury associated with head‐first impact (HFI). However, CFD has not been reliably reproduced in experimental or numerical studies, limiting understanding of its underlying mechanics. This study quantified the influence of preimpact head‐forward posture on lower‐neck sagittal‐plane loading associated with CFD during HFI.

**Methods:**

A detailed finite element head–neck model was enhanced and evaluated against literature data, then used to investigate HFI response at a 2 m/s vertical drop impact velocity. A reposturing procedure achieved graded head‐forward eccentricity from 0 to 50 mm in 5 mm increments, reflecting postures observed during anticipatory neck bracing. A matched cadaveric HFI experiment was subsequently performed with 30 mm eccentricity.

**Results:**

Simulations demonstrated that eccentricities of 15 mm or more produced an S‐shaped neck deformation during impact, as observed in prior experiments that produced CFD, accompanied by marked increases in anterior intervertebral shear and flexion bending at C7/T1. The matched cadaveric experiment produced bilateral C7/T1 dislocation, consistent with the predicted loading and deformation trends. However, complete CFD was not reproduced computationally because current intervertebral soft‐tissue failure formulations did not capture the observed failure progression.

**Conclusions:**

Preimpact head‐forward eccentricity can substantially increase lower‐neck loading and susceptibility to CFD under the inverted‐drop conditions studied.

## 1 Introduction

Subaxial cervical facet dislocation (CFD) is a severe neck injury that most often occurs during vehicle rollovers, falls, and dives into shallow water [1, 2]. During these head‐first impact (HFI) events, the body is inverted and the arrested head motion causes the torso to transfer a downward inertial load through the cervical spine [3]. Despite this established causal link between HFI and CFD, replicating the injury in HFI experiments or computational human models has proven challenging. The lack of repeatable dynamic models of subaxial CFD highlights the current gaps in understanding its underlying mechanisms, thereby hindering the advancement of effective injury prevention strategies and devices [3].

Inverted drop head–neck component testing has been widely adopted as the preferred experimental method for investigating cervical spine injury mechanisms associated with HFI [3]. In this method, head–neck human cadaver specimens are rigidly fixed in an inverted orientation to the underside of a weighted drop‐tower carriage (typically 16 kg, representing the upper torso of a 50th percentile male [4]). The carriage is then released from a controlled height to generate a head‐first impact sufficient to induce cervical spine injury. This method offers a simplified and controlled experimental setting, enabling systematic variation of key input parameters and their effects on injury patterns (e.g., Nightingale et al. [4]).

CFD has been sporadically observed during inverted drop HFI tests, but the precise experimental and specimen‐specific factors influencing its occurrence remain unclear [3]. Bilateral CFD (C6/C7) was produced in 2 of 22 specimens during a study that explored the effect of varying impact surface parameters on cervical spine mechanics and injuries during HFI [4, 5]. However, these injuries occurred under disparate experimental conditions, including a padded horizontal surface and a rigid surface angled at 15°, and the precise preimpact head–neck postures were not reported. Notably, these experiments also described dynamic neck buckling that produced “S‐shaped” deformation, with local lower‐neck flexion associated with the bilateral CFD specimens [5].

Anterior intervertebral shear, typically occurring in combination with local lower‐neck flexion during dynamic neck buckling, has been repeatedly associated with CFD across experimental and computational studies [4, 5, 6, 7, 8, 9, 10, 11, 12]. This combined loading mechanism has also been demonstrated in an inertial functional spinal unit model, in which subaxial CFD occurred under anterior shear and flexion loading [13]. However, that whiplash‐type configuration excluded the head and superior cervical levels and used an incremental‐trauma protocol and therefore did not reproduce the loading pathway associated with HFI.

More directly relevant to head‐forward posture, highly controlled, low‐rate experimental studies demonstrated that vertical compression of head–neck specimens with anterior head protrusion and a horizontal Frankfort plane (FP) produced the S‐shaped or “ducking” neck posture, generating lower‐neck intervertebral flexion and anterior shear loads and motions sufficient to cause CFD [6, 14]. Although the boundary and loading conditions of these quasi‐static experiments likely diverge from the dynamics of real‐life HFI events, they identify a plausible lower‐cervical loading pathway by which an eccentric head‐forward posture (HFP) may elevate the likelihood of CFD in actual HFI scenarios. Such HFP during HFI may arise from inversion, with or without reflexive neck muscle bracing, which was shown in an experimental study of 11 human volunteers to produce an average anterior head eccentricity of 26 mm relative to the line of action of torso inertia [15]. Such volunteer data suggest that bracing‐related head‐forward posture during HFI could plausibly span neutral alignment through typical offsets of approximately 26 mm, and in some cases extend to larger eccentricities under more extreme bracing or age‐related postural variation.

The influence of preimpact sagittal‐plane head eccentricity on cervical spine injury mechanisms has previously been described in the dynamic experiments of Pintar et al. [10] and Maiman et al. [16], which suggested that anterior eccentricities of 20–70 mm increase the risk of severe lower cervical injury, including CFD. However, those studies used piston‐driven impacts applied to preflexed head–neck preparations, with eccentricity altered through sagittal‐plane rotation of the cervical column rather than isolated anterior head translation. These conditions differ from the head–neck posture associated with bracing‐related head‐forward posture [15], from the S‐shaped deformation produced with a horizontal Frankfort plane that can lead to CFD [6, 14], and from the inverted‐drop loading pathway by which impact energy is transferred through the head–neck complex during HFI [4]. A systematic investigation is needed to isolate the effect of anterior head translation on lower neck mechanics under inverted‐drop HFI conditions.

Advanced finite element (FE) human body models (HBMs) allow controlled evaluation of how HFI parameters alter cervical loading conditions in ways that are impractical to isolate experimentally [17]. FE models can be configured to precisely replicate HFI experimental conditions, enabling systematic analyses of how HFI parameters influence cervical spine mechanics under controlled conditions. Various HFI simulation studies have shown that factors such as impact surface properties, velocity, preimpact flexion, muscle activity, and torso‐end boundary conditions affect neck response [8, 18, 19, 20, 21]. However, a systematic investigation of pre‐HFI anterior head eccentricity under inverted‐drop conditions has not previously been performed. Such an analysis complements previous work by isolating the effect of a posture parameter that varies during real‐world HFI and by examining its influence on lower‐neck loading patterns associated with CFD.

The overall aim of this research was to investigate the effect of head‐forward posture on lower neck loading associated with CFD during HFI. It was hypothesized that a preimpact forward head posture would elevate lower‐neck shear force and flexion moment, thereby increasing CFD susceptibility. The specific aims were to: (1) enhance an existing FE head and neck model to improve its suitability for inverted‐drop HFI simulation; (2) identify the effect of preimpact head eccentricity on lower‐neck loading and kinematics during HFI simulations; and (3) compare the simulation‐predicted response with a matched cadaveric HFI experiment to assess how well the model reproduced the targeted loading and deformation mechanism.

## 2 Methods

**FIGURE 1:**
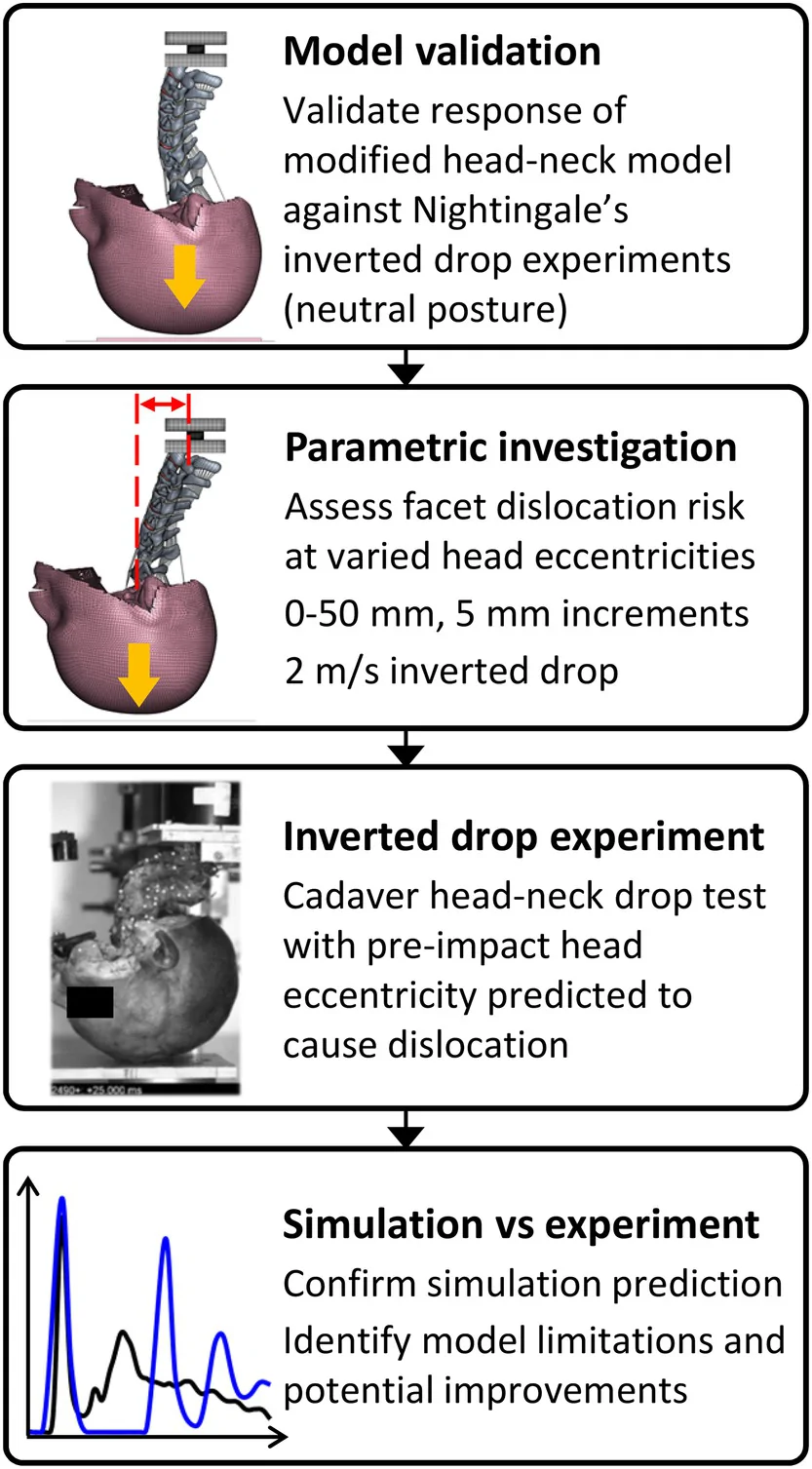
Overview of study approach.

This study comprised four sequential stages (Figure 1). A detailed head–neck HBM was first enhanced and validated for inverted drop HFI by comparing dynamic FE impact kinetics to published cadaveric component data. The model was then used to conduct parametric simulations that systematically varied pre‐HFI head eccentricity to assess its effect on lower‐neck loading and associated CFD risk. Based on these results, an inverted drop experiment was designed using a cadaveric head–neck specimen to test a high‐risk posture identified by the simulations. Experimental and simulation outputs were then compared to assess how well the model reproduced the targeted loading and deformation mechanism and to identify limitations relevant to future model development.

### 2.1 Head–Neck FE Model Setup and Enhancements

**FIGURE 2:**
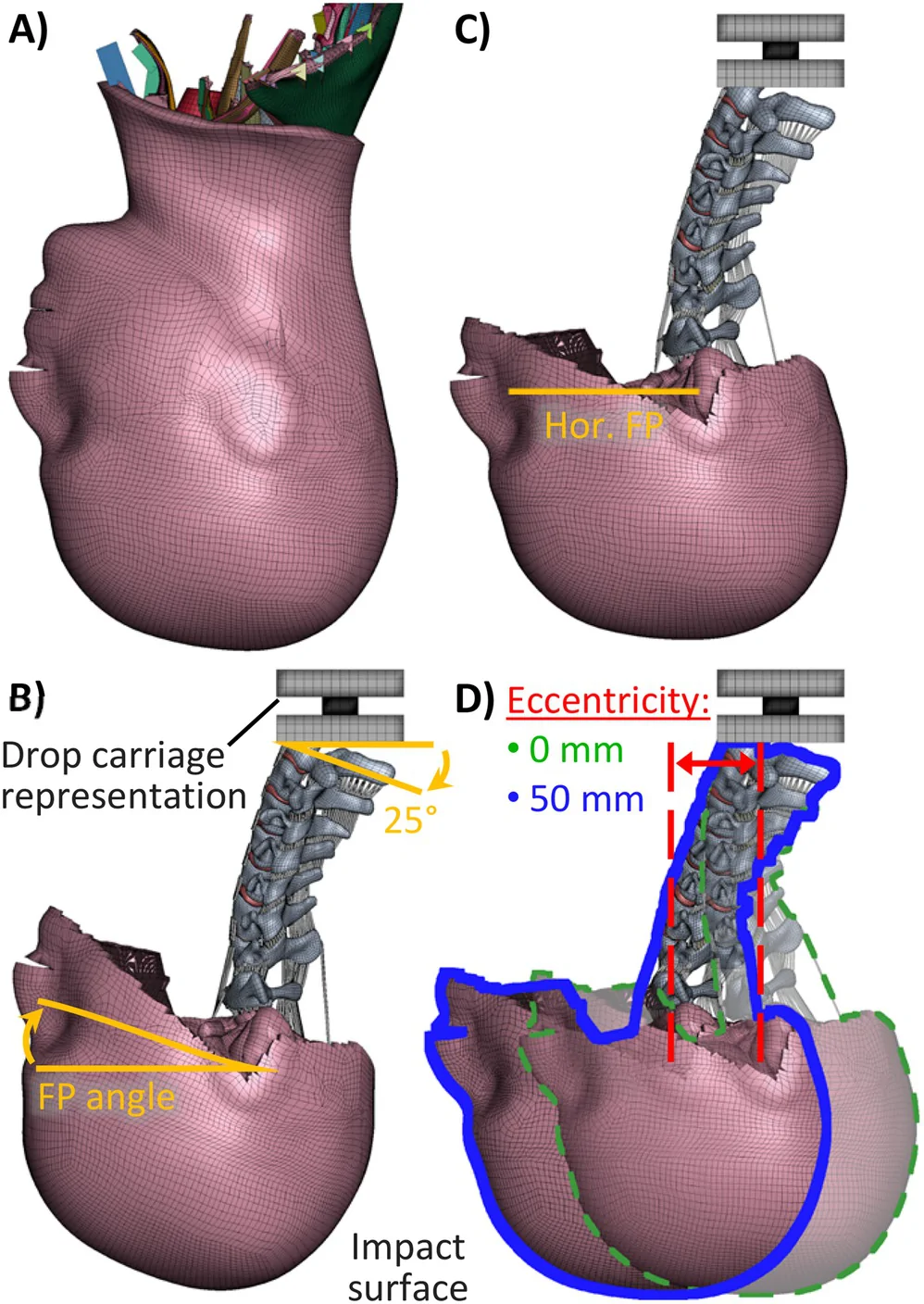
Modeling overview: (A) Baseline Global Human Body Models Consortium 50th percentile male detailed head–neck model. (B) Stripped and rotated model attached to a geometric representation of the drop carriage and positioned directly above impact surface to replicate prior drop experiment setups. (C) Preimpact model reposturing to achieve a horizontal Frankfort plane (FP) and vary anterior head eccentricity between 0 and 50 mm (D).

Inverted drop HFI was simulated using a modified version of the Global Human Body Models Consortium 50th percentile male detailed head–neck model (GHBMC M50‐HN, Version 6.0). To replicate the conditions of previously published inverted drop head–neck component experiments [4] and simulations [18], the neck flesh, all musculature, tendons, hyoid bone and mandible, and all attachments, were removed and the entire model was rotated so the T1 superior endplate was 25° to the horizontal plane (Figure 2A,B). Pilot simulations were conducted to assess the suitability of the baseline model setup for low‐velocity head‐first impact onto a rigid plate and to address early‐phase response artifacts that confounded interpretation of neck loading [18, 20]. As described in Supporting Information S1, scalp and skull definitions were iteratively updated to reduce excessive head rebound and improve agreement with published inverted‐drop force‐time histories. These updates included replacing the scalp material properties with those used in the Total HUman Model for Safety HBM (THuMS, v4.02, Toyota, Japan) [22], removing duplicated skull cortical shell layers that artificially increased stiffness, and reducing the elastic modulus of the skull cortical solid elements (15–10 GPa). Consistent with Darweesh [18], the pilot simulations produced C0 fractures that do not occur during HFI experiments at impact velocities < 3 m/s [4]. Bone erosion was therefore disabled to avoid nonphysiological brittle cranial fracture within the energy range relevant to the drop tests and to minimize unrealistic load transfer to the neck that could confound the soft‐tissue mechanisms most relevant to CFD.

### 2.2 Preimpact Head–Neck Reposturing

Each simulation comprised initial model reposturing to achieve the desired head–neck pose prior to impact (Figure 2C,D). T1 motion was temporarily constrained, and the desired rotation and translation were imposed to a virtual skull node over 0.5 s (evenly split between rotation and translation). To standardize cranial orientation while preserving physiological cervical alignment, the GHBMC head was rotated (extension) by 4.96° about its physiological center of rotation at the atlanto‐occipital joint [23, 24] to achieve a horizontal Frankfort plane; intervertebral joint motions were not kinematically constrained during prepositioning, allowing the cervical spine to settle into a compatible posture. The head was then translated horizontally (along the anterior–posterior axis of the Frankfort plane) to produce the desired eccentricity, defined as the horizontal distance between the center of the foramen magnum and the posterior‐inferior corner of T1 (Figure 2D). Additional damping was applied to the entire head–neck model throughout reposturing to obviate unwanted dynamic responses. The damping was then removed, and an additional 10 ms settling period (no imposed motion) was applied. The stresses, vertebral motions, and deformations of the head–neck assembly associated with the reposturing were retained in the model, and the drop was simulated by imposing the desired vertical impact velocity.

### 2.3 Inverted Drop Simulations With Head–Neck Model

The inferior portion of T1 was rigidly connected to a simplified representation of an instrumented 16‐kg drop carriage (Figure 2B). To simulate a typical drop rail, the simplified carriage assembly was constrained to vertical translation only. The head was positioned just above a rigid surface representing the impact plate of the drop tower and HFI was simulated by assigning an initial downward velocity to the head–neck model.

Three‐dimensional coordinates of key anatomical landmarks (predefined virtual nodes) on the skull and each vertebra, and six degrees of freedom loads for the impact surface, drop carriage, and intervertebral joints, were extracted from the simulations at 100 kHz. Forces acting on the impact surface were obtained directly from the rigid‐wall kinematical constraint used to model this surface, while forces and moments on the drop carriage were extracted from a predefined cross‐section of the solid part representing the upper load cell. Similarly, intervertebral loads were derived from a cross‐section through the inferior vertebra of each level and were output in a local coordinate system that moved with each vertebra [25].

Extracted data were processed using custom MATLAB code (R2021b, Mathworks, MA, USA). Fourth‐order, two‐way, low‐pass Butterworth filters were applied to the data per SAE J211 [26]. At each timepoint, anatomical coordinate systems (ACS) were defined for the cephalus and each spinal level [25]. Three‐dimensional global and segmental kinematics were calculated by deriving Euler angles and linear translations from affine transformations between ACS pairs at each simulation step [27].

### 2.4 Model Validation for HFI With a Neutral Neck Posture

The modified GHBMC model's response to inverted drop, with a neutral pre‐HFI head–neck posture, was initially validated through comparison against existing experiments available in literature. The boundary and input conditions for relevant tests from [4]/1997 HFI study (N22, N24, N26) [4, 5] were replicated in HFI scenarios simulated using the modified head–neck model. Preimpact head eccentricity was “anatomically neutral” (0 mm) and a 3 mm‐thick Teflon sheet was modeled on top of the impact plate using solid elements with published material properties obtained from the literature [28]. The average impact velocity of the three relevant specimens (2.96 m/s) was applied to the head–neck model.

The model outcomes were validated against the Nightingale experimental results, including HFI kinematic progression and head‐ and torso‐end kinetics. These comparisons served to confirm that the model produced reasonable head impact loads and that the timing and magnitude of subsequent neck loading during the carriage loading phase were consistent with prior experimental observations. The neck and impact surface resultant force traces were extracted from Nightingale et al. [5] and were synchronized with the corresponding simulation outcomes for qualitative and quantitative comparisons. Intervertebral motion data from these experiments was not reported, so kinematic simulation outcomes were not analyzed.

### 2.5 Parametric Investigation of Preimpact Head Eccentricity on Head–Neck HFI Response

For the parametric investigation simulations, pre‐HFI forward head eccentricity was systematically varied from 0 to 50 mm in 5 mm increments. The coefficient of friction of the rigid impact surface matched the experimental rig used in the subsequent experiments (*μ* = 0.52); this was calculated from in‐house experimental friction tests between human scalps and the drop‐tower impact plate (see Supporting Information S1). An initial impact velocity of 2 m/s was applied; this is within the low‐to‐moderate range used in prior HFI experiments and has been associated with cervical injury under nonneutral head–neck postures [10, 29]. Each simulation HFI response, including global and intervertebral kinetics and kinematics, was analyzed qualitatively and quantitatively. The relative risk of CFD across simulations was then assessed based on these biomechanical responses.

### 2.6 Experimental HFI With Preimpact Head Eccentricity

**FIGURE 3:**
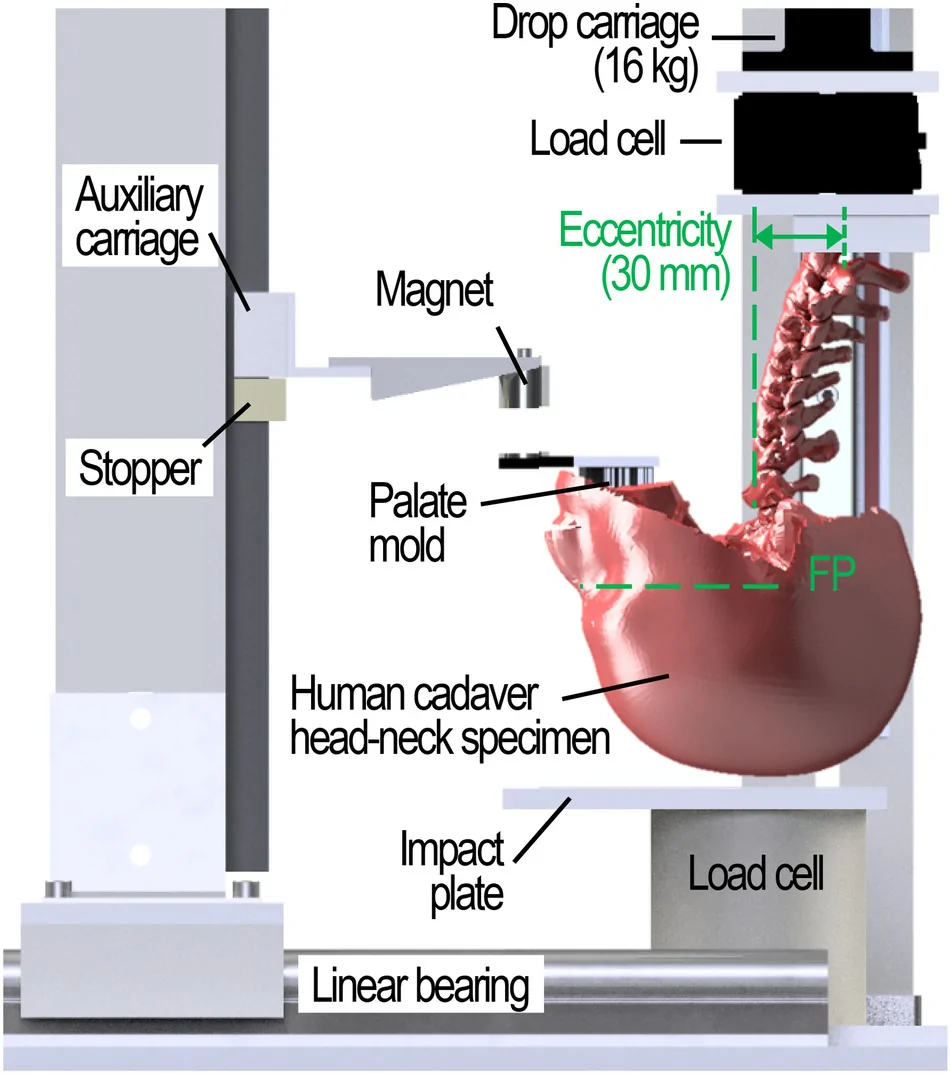
Schematic of the human cadaver head–neck component inverted drop experimental setup.

To assess the simulation prediction for HFI with a preimpact HFP, a fresh‐frozen human cadaver osteoligamentous head–neck (cephalus‐T1, male, 70 years old) underwent inverted drop HFI testing using a drop tower (HREC approval H‐2023‐098); the FP was horizontal and head eccentricity was 30 mm (Figure 3). The 16 kg carriage was released from a height of 240 mm to achieve a 2 m/s head‐impact velocity. Head–neck posture was maintained during the drop via an auxiliary parallel drop rail; the auxiliary carriage interfaced with the head via a specimen‐specific, 3D‐printed hard palate mount that ensured a horizontal FP. Head constraints were removed immediately prior to impact via external‐trigger release of an electromagnet that connected the palate mount and the auxiliary carriage. Impact surface and caudal loads were measured by 6‐axis load cells and carriage position was measured with a linear encoder (both 50 kHz). Synchronized stereo high‐speed camera footage (10 kHz; Phantom VEO1010, Vision Research, USA) was captured for qualitative kinematic analysis and to identify the timepoint of tissue damage identified via posttest dissection and CT scan (NAEOTOM Alpha, Siemens, Munich, Germany; 0.2 mm isotropic voxels).

## 3 Results

All 12 simulations (1 for Nightingale comparison and 11 for the parametric analysis) were successfully solved using the commercial explicit finite element code ANSYS LS‐DYNA (R14.0‐515, MPP, Double precision) with 16 compute cores on the Phoenix High‐Performance Computing (HPC) platform at The University of Adelaide. Each initial reposturing simulation required approximately 12 h of compute time, while the subsequent HFI simulation completed in up to 3 h.

### 3.1 Model Validation for HFI With a Neutral Neck Posture

**FIGURE 4:**
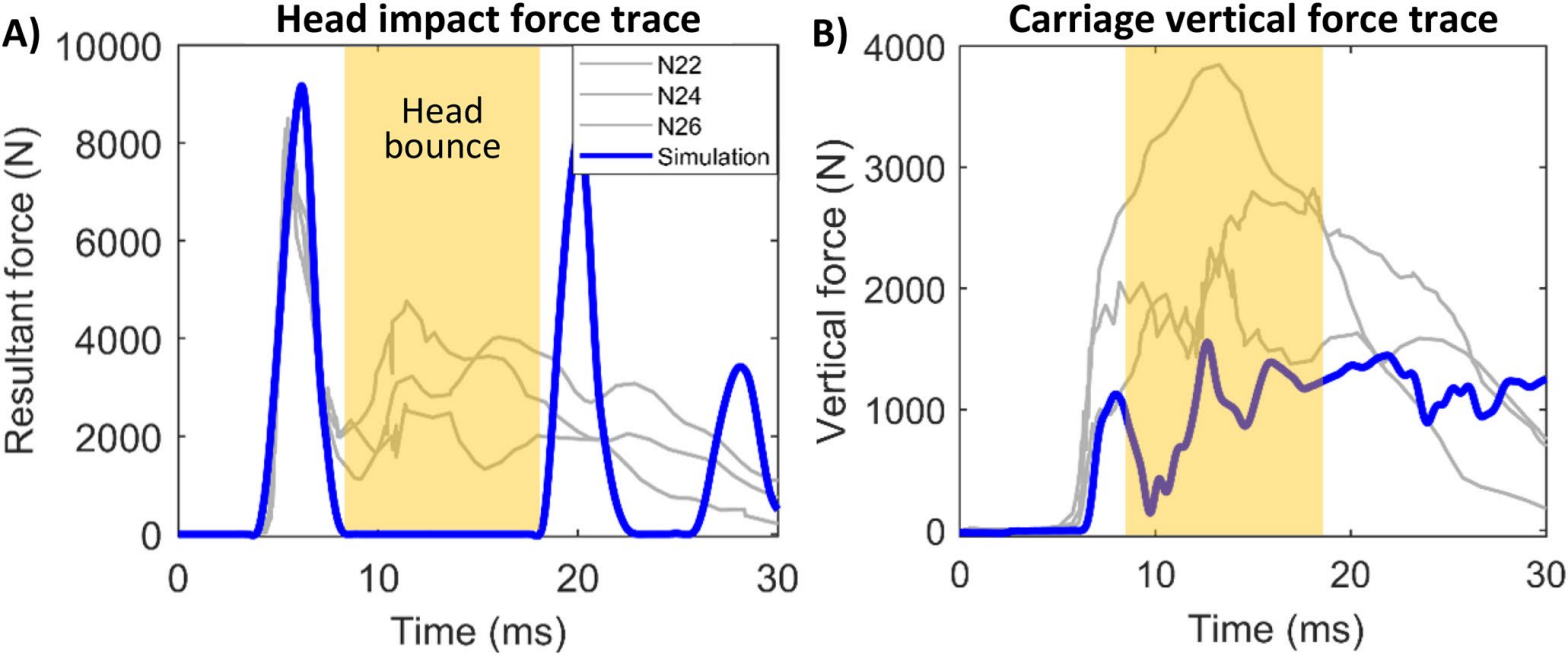
Comparison of simulation and available experimental data from Nightingale et al. [5] for: (A) head impact force; and (B) carriage vertical force.

The initial head loading phase of the simulations that replicated the Nightingale flat rigid plate HFI experiments [5] closely matched the force trace data for the corresponding specimens (Figure 4A, Table 1). Modifications to the scalp and skull material definitions helped to reduce the considerable head bouncing in the original model (Supporting Information S1), but the simulations still exhibited some residual post‐HFI head bounce (Figure 4A). While unloading of the impact surface (head bounce) occurred in the experiments, complete rebound was not observed in the experimental data (i.e., impact surface force not dropping to 0 N). The excessive head bounce in the simulation resulted in loss of axial loading of the neck by the carriage between 4 and 7 ms postimpact, and a slight reduction in overall magnitude of axial neck load (Figure 4B). Nonetheless, peak neck force and time‐to‐peak (Table 1) as well as temporal evolution of the simulated force trace profile were comparable to the available experimental data. Importantly, the force‐time responses of the simulation and experiments converged after ~12 ms post‐HFI, when neck loading was dominated by the carriage and when HFI‐related neck injury typically occurs [5].

**TABLE 1:** Experimental data published by Nightingale et al. [5] and corresponding head impact and neck force metrics from the equivalent simulation.

	Peak head impact force (kN)	Slope of head impact force rise time (kN/ms)	Peak vertical neck force (kN)	Time from head impact to peak neck force (ms)
*N22*	8.0	10.0	2.0	12.6
*N24*	8.5	11.2	1.8	7.9
*N26*	7.8	10.3	3.9	7.5
**Nightingale** **[Avg (SD)]**	**8.1 (0.4)**	**10.5 (0.6)**	**2.6 (1.2)**	**9.3 (2.8)**
**GHBMC**	**9.2**	**5.1**	**1.6**	**6.5**

*Note:* Mean experimental values and corresponding simulation outputs are bolded for direct comparison.

### 3.2 Parametric Investigation of Pre‐HFI Head Eccentricity

**FIGURE 5:**
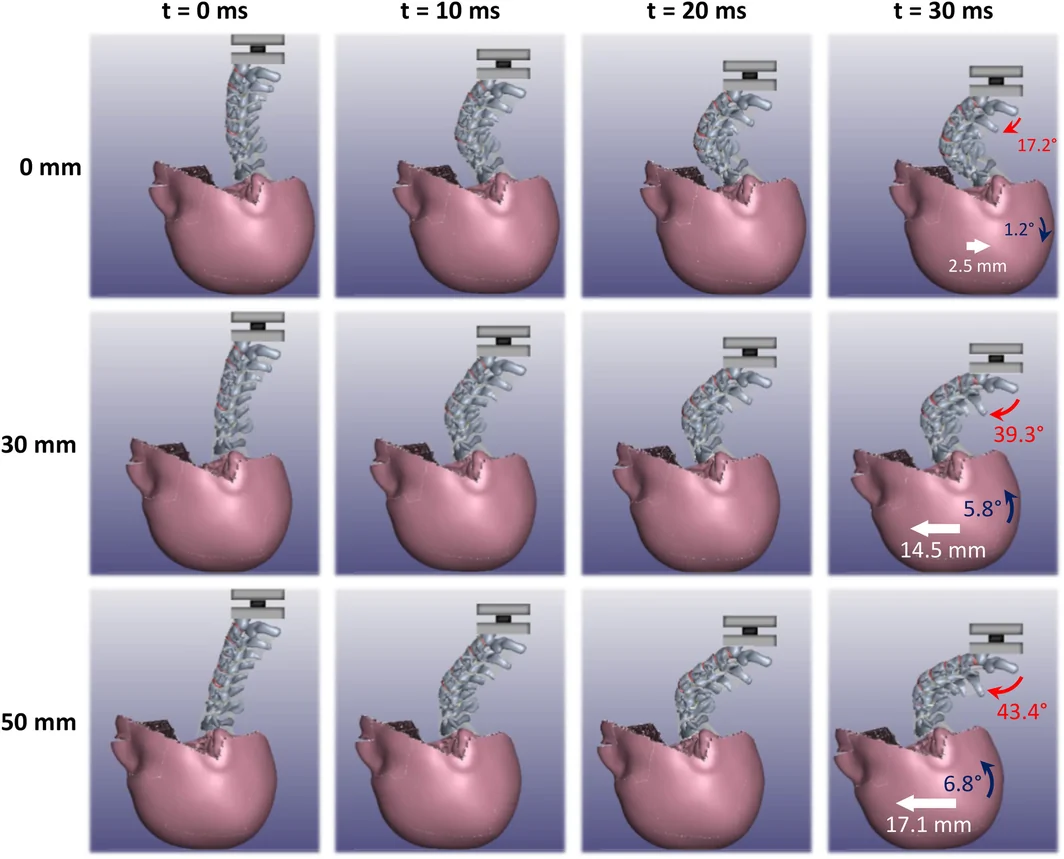
Simulation progressions (0–30 ms) following head‐first impact for 0, 30, and 50 mm preimpact head eccentricities. C7/T1 rotation angle (red), and head rotation (blue) and translation (white) are indicated at *t* = 30 ms.

**FIGURE 6:**
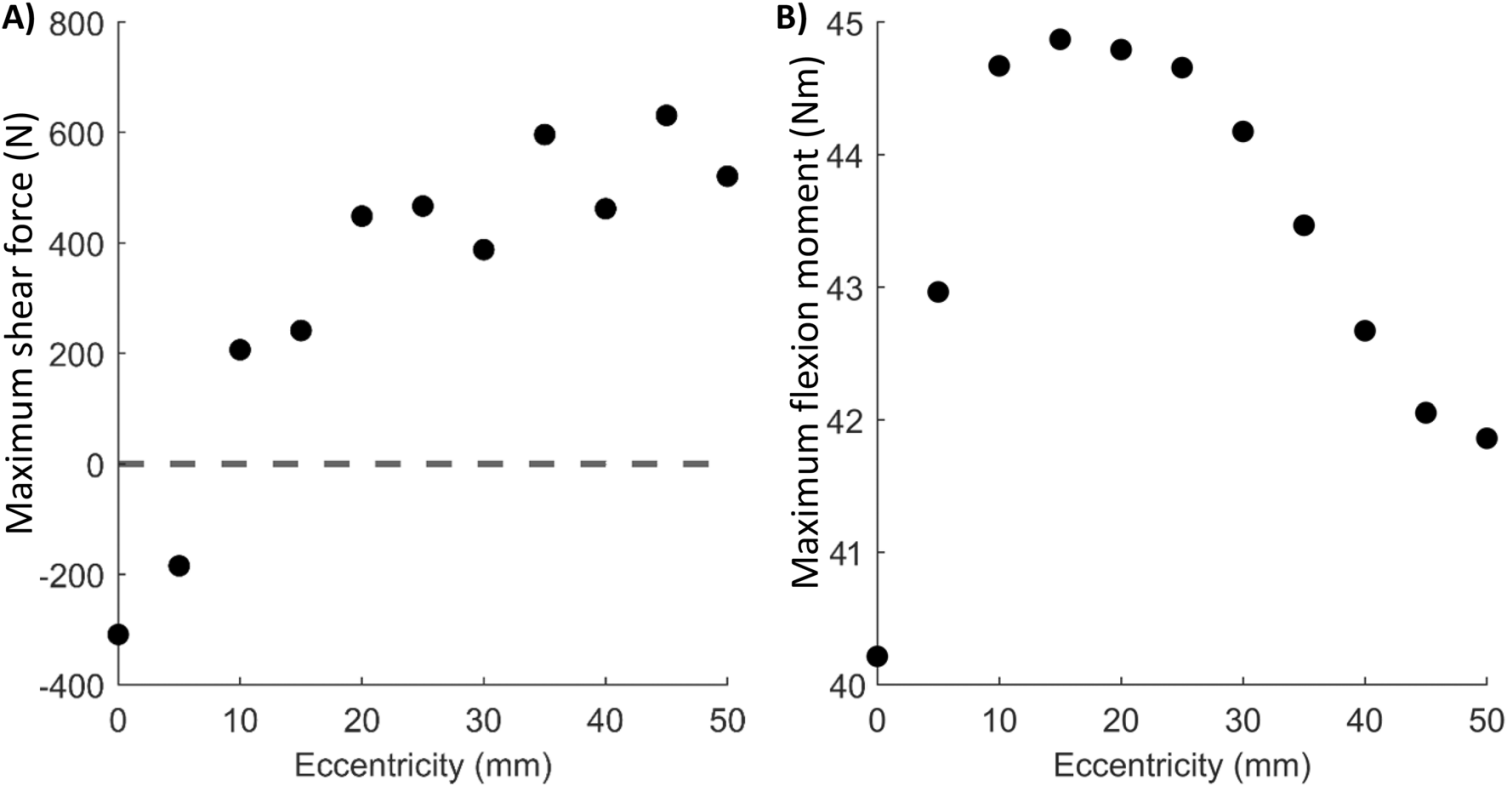
Peak C7/T1 shear force (A) and flexion moment (B) during the carriage loading phase versus preimpact head eccentricity.

Increasing head eccentricity while maintaining a horizontal Frankfort plane led to a reduction in cervical lordosis, effectively straightening the neck before impact (Figure 5, *t* = 0 ms). Initial impact responses were consistent across simulations, with all producing peak vertical impact forces between 5.2 and 5.9 kN, occurring approximately 2 ms post‐HFI (Supporting Information S2, Figure S3A). All simulations exhibited a transient unloading interval after the initial pulse, with head impact force returning to approximately 0 N and contact re‐established by ~13 ms. Subsequent carriage compression then induced the characteristic S‐shaped neck posture, which became more pronounced with increasing pre‐HFI head eccentricity (Figure 5, *t* = 10–30 ms). This posture‐dependent curvature response was associated with head (C0–C2) extension rotation and forward translation, inducing supraphysiologic C7/T1 flexion rotation (Figure 5) and progressively greater lower‐neck sagittal‐plane loading during the carriage‐loading phase (Figure 6).

Differences in head–neck curvature during the carriage loading phase corresponded to distinct differences in lower‐neck sagittal‐plane loads (Supporting Information S1, Figure S2C). In simulations with pre‐HFI eccentricity of 15 mm or less, C7/T1 shear forces were posteriorly directed or near zero (< 200 N). In contrast, simulations with eccentricity exceeding 15 mm exhibited larger flexion moments and substantial anterior shear forces, with peaks reaching levels theoretically sufficient to induce CFD (~600 N; Quarrington et al. [11]). Peak anterior shear increased sharply up to about 15 mm and then plateaued, while peak flexion moment peaked near 15 mm and decreased at eccentricities over 30 mm (Figure 6). To accommodate potential experimental variability and ensure a high likelihood of inducing CFD, a pre‐HFI eccentricity of 30 mm was chosen for the validation experiment.

### 3.3 Experimental HFI With Preimpact Head Eccentricity

**FIGURE 7:**
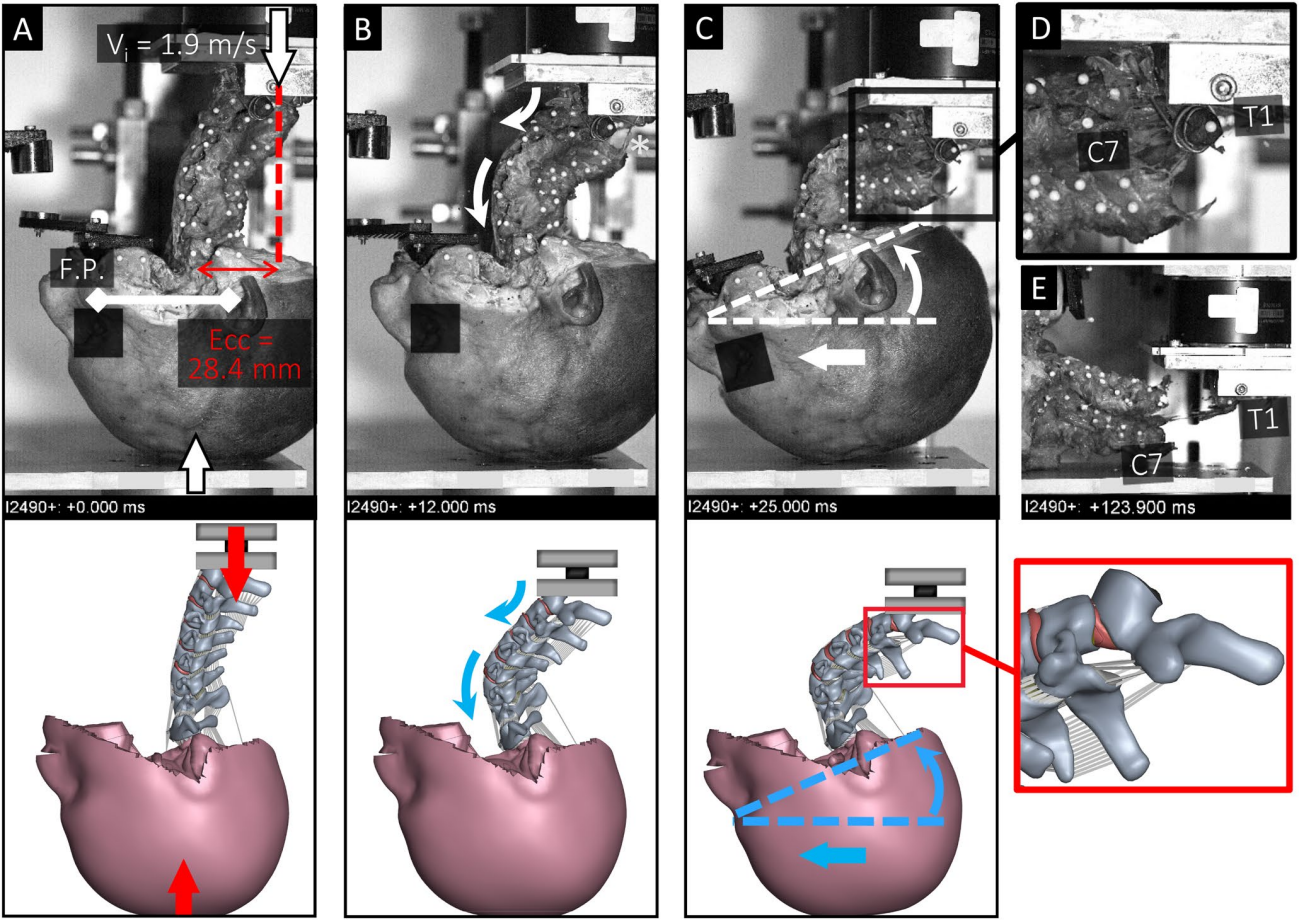
High‐speed video footage from the inverted drop experiment (top row) and outputs from the corresponding simulation (bottom row); key loads and motions are indicated by the arrows. (Panel A) At the point of impact (*t* = 0), the head was arrested with a horizontal Frankfort plane (F.P.) and an eccentric posture (Ecc = 30 mm). (B) After ~12 ms, carriage compression with minimal head motion produced an S‐shaped neck and complete rupture of the C7/T1 interspinous ligament (ISL) in the experiment (*) but not the simulation. (C) At 25 ms postimpact, further torso compression, plus head extension and anterior translation, causes complete rupture of all C7/T1 soft‐tissues (D), and complete dislocation (E). However, in the simulations, the intervertebral disc, bilateral facet capsules, and deepest ISL elements of the model remained intact, preventing dislocation.

The experimental setup subjected the specimen to inverted HFI with preimpact eccentricity of 28.4 mm, an FP angle of −1.3°, and impact velocity of 1.9 m/s. The HFI progression closely mimicked the simulation with pre‐HFI eccentricity of 30 mm (Figure 7). As expected, the supraphysiologic C7/T1 intervertebral loads and motions produced during the carriage loading phase caused total rupture of all C7/T1 intervertebral soft tissues, resulting in bilateral CFD.

**FIGURE 8:**
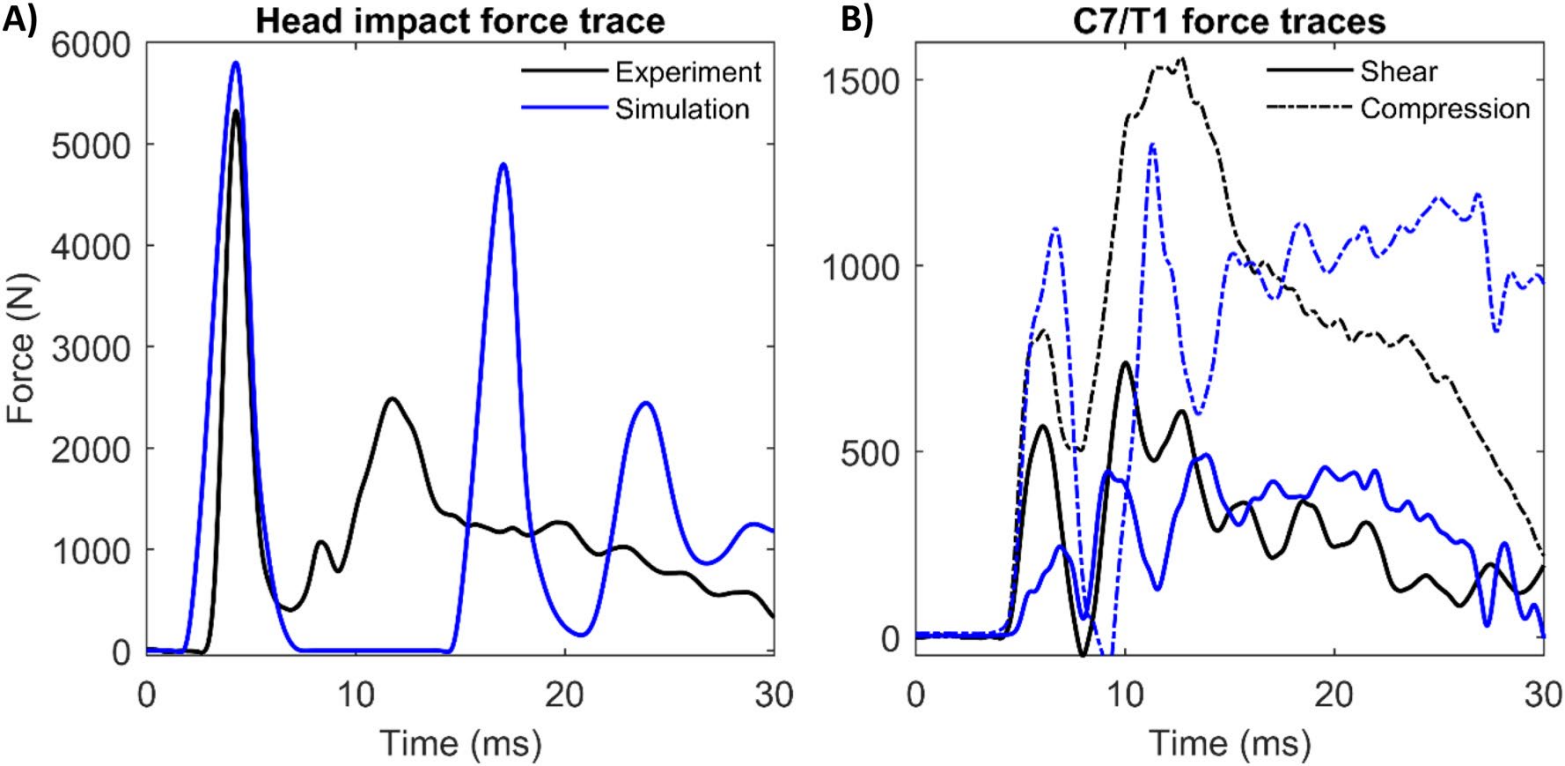
Comparison of simulation to matched experiment data for (A) head impact and (B) C7/T1 shear and compression forces.

The head‐ and torso‐end experimental force data showed good agreement with the corresponding simulation outputs (Figure 8). Peak vertical forces during the initial head loading phase were comparable (5.3 kN [experiment] vs. 5.8 kN [simulation]), though the excessive head rebound observed in the simulations was not present in the experiment. Like the Nightingale simulations, head bounce in this simulation reduced axial load measured by the carriage immediately postimpact, resulting in a slightly lower overall magnitude of neck loading compared to experimental data during this phase (7–12 ms, Figure 8B). However, the temporal evolution of C7/T1 shear and compression force traces, as well as intervertebral kinematics in each region of the cervical spine (Supporting Information S3, Figure S4), remained highly similar until the onset of experimentally observed C7/T1 soft‐tissue failure. Specifically, complete rupture of the interspinous ligament (ISL) occurred at ~9–10 ms postimpact, followed by sequential failure of the remaining intervertebral soft tissues, which corresponded to the force drops observed in the experimental data (Figure 8B). In the corresponding simulation, complete erosion of the C7/T1 ISL did not occur because the deepest elements did not reach their failure threshold, and the intervertebral disc (IVD) and facet capsular ligaments (CL) also remained intact, preventing CFD from occurring.

## 4 Discussion

This study systematically investigated the effect of preimpact head‐forward posture on the risk of subaxial CFD during head‐first impacts, building upon observations from quasi‐static experiments that this head–neck posture produces intervertebral shear and flexion loads in the lower neck which may lead to CFD [6, 14]. Computer simulations showed that increasing pre‐HFI head eccentricity progressively reduced cervical lordosis before impact and promoted a more pronounced S‐shaped deformation during carriage loading. This posture‐dependent curvature response was associated with a marked increase in C7/T1 anterior shear, accompanied by an elevated flexion moment, around 15 mm of eccentricity. These findings informed the design of an inverted drop experiment, in which a human cadaveric head–neck specimen prepositioned with 30 mm of anterior head eccentricity sustained bilateral C7/T1 CFD, providing experimental confirmation of the simulation predictions.

The relationship between flexion moment and anterior shear at C7/T1 changed character as eccentricity increased. At small eccentricities the lower neck was loaded predominantly in flexion, and shear force was small or posteriorly directed; as eccentricity increased and the neck adopted a more pronounced S‐shaped deformation, anterior shear rose steeply and then plateaued, whereas the flexion moment peaked near 15 mm and declined at larger eccentricities (Figure 6). The escalation of dislocation risk above 15 mm therefore followed anterior shear rather than flexion moment, consistent with a localized flexion‐and‐shear mechanism at the buckled lower‐cervical segment rather than progressive sagittal bending of the head and neck as a whole. In this sense the flexion moment marks the developing buckled configuration, whereas the anterior shear it generates is the proximate determinant of dislocation.

Computational models offer a powerful means to investigate injury mechanisms systematically and gain insights that are impractical to obtain through experiments alone. A major limitation of prior inverted drop HFI experimental studies has been the inability to precisely control preimpact head–neck posture, resulting in sporadic and study‐specific reports of CFD [4, 5, 30]. The present FE simulations addressed this limitation by parametrically varying head eccentricity to evaluate its influence on CFD risk in a controlled and repeatable manner, without the need for extensive cadaveric testing. The high‐risk posture identified through simulation, which would have required at least 11 precisely controlled cadaveric tests to isolate experimentally, was subsequently tested and confirmed in a single inverted drop experiment.

The parametric investigation indicated a likely increase in C7/T1 injury risk beyond 15 mm of pre‐HFI eccentricity, resulting in loads and motions that exceeded physiological thresholds [11, 31]. This result supports the hypothesis of Pintar et al. [10] that HFI‐related CFD is associated with a C0‐T1 eccentricity of 31 mm. A horizontal FP was not maintained in those tests, but the authors of the experimental series suggested that smaller (< 20 mm) and larger eccentricities (> 70 mm) are associated with a reduce risk of severe neck injury from HFI due to the increased ability of the neutral spine to withstand axial compression, and the tendency for preflexed head–necks to adopt a hyperflexed (chin‐to‐chest) posture [10, 16]. These hypotheses could be investigated in future experiments using the inverted drop HFI model developed in the current study.

The present findings highlight the sensitivity of lower‐neck loading to preimpact head–neck posture under inverted‐drop HFI conditions, but the translational relevance must be considered in light of the study limitations. The inverted drop model constrains the torso through a rigid mounting system, which may not fully replicate the inertial and kinematic responses of the full body during real‐world impacts [32]. Notably, C7/T1 injuries have been difficult to reproduce in inverted‐drop setups, supporting the interpretation that the imposed initial conditions substantially contributed to the observed CFD. Accordingly, we interpret these results as identifying a high‐risk posture for elevating lower‐neck shear under this boundary condition, rather than characterizing the full spectrum of clinical CFD patterns.

The absence of passive and active musculature limits the fidelity of neuromuscular contributions that may influence both load transmission and injury thresholds, particularly in HFI scenarios where reflexive or anticipatory muscle activity may contribute to neck injury mechanics [33]. Although eccentricity and head rotation were prescribed by standardizing head orientation to a horizontal Frankfort plane, small residual differences in cervical alignment may still influence segment‐level mechanics because intervertebral joints were intentionally left unconstrained during prepositioning to allow the spine to settle into a compatible posture.

The generalizability of the experimental findings is limited by the use of a single cadaveric specimen and inter‐individual variability in cervical tissue structure and properties. The experimental specimen was 70 years old and screening did not indicate excessive cervical degeneration. Many of the cervical soft‐tissue property datasets used to inform head–neck HBMs are derived from older adult cadaver tissue, and experimental studies demonstrate measurable age effects on cervical ligament stiffness and failure properties [34, 35]. Nonetheless, age‐stratified, segment‐specific and combined‐loading failure data remain limited, particularly under dynamic conditions relevant to head‐first impact, so age‐related influences on failure pathways cannot be fully resolved [36]. Finally, consistent with the scope of this work, the simulations were used to guide selection of an eccentricity likely to elevate lower neck loads for subsequent experimental testing, rather than to predict specimen‐specific failure; additional specimens across age and degeneration spectra are needed to confirm generalizability.

Comprehensive quantitative biofidelity assessment (e.g., CORA, ISO) was outside the scope of this posture‐focused study, but comparison of the experimental and simulated responses highlighted specific deficiencies in the HBM's replication of injury‐relevant neck mechanics under HFI, guiding future model refinements and accelerating progress toward more accurate simulations of neck injury. Intervertebral rotations at the upper (C2/C3), middle (C5/C6), and lower (C7/T1) cervical spine during the simulated HFI were broadly consistent with the experiment up to about 9 ms postimpact (Supporting Information S3, Figure S4), when posterior ligament failure at C7/T1 occurred in the experiment and produced a rapid, supraphysiologic increase in flexion rotation. Although the simulations demonstrated intervertebral shear forces sufficient to rupture all intervertebral soft tissues, current failure criteria limitations for the ISL, CL, and IVD prevented CFD from occurring. Importantly, the comparison with the matched experiment identified specific limitations of the current GHBMC intervertebral soft‐tissue response to HFI, thereby helping define the model developments needed before tissue‐level injury metrics and multi‐level internal load distributions can be interpreted with confidence.

These limitations are evident in how failure is currently represented in the GHBMC detailed head–neck HBM. The model only incorporates endplate avulsion as an injury criterion for the IVD, excluding compression‐induced herniation and physical shearing, mechanisms expected during CFD [37]. Additionally, progressive ligament failure definitions were derived from pure tensile displacement testing of bone‐ligament‐bone specimens and implemented as tension‐driven, fiber‐aligned criteria [38], which do not fully represent multi‐axial failure behavior under combined compression, shear, and bending. Failure mechanics are also influenced by loading direction, as demonstrated for the knee's anterior cruciate ligament [39], which likely makes pure tensile, fiber‐aligned criteria inadequate for ligaments such as the ISL and CL that experience complex, multi‐axial loading. In the GHBMC, the ISL is modeled as parallel fibers, with progressive failure thresholds increasing from outer to deep fibers. However, the matched experiment in the current study showed that ISL failure accompanying CFD was driven by intervertebral flexion, which inherently causes greater displacement of the outer ISL fibers. Consequently, the corresponding simulation predicts failure of outer fibers but preserves deep fibers, preventing CFD from occurring. Because the current intervertebral soft‐tissue failure formulations do not capture the multi‐axial failure progression observed experimentally, tissue‐level stress/strain outputs and multi‐level internal joint load predictions were not interpreted as injury risk metrics in this study. These limitations likely contributed to the model's underprediction of intervertebral soft tissue failure, compared to the experiment, and the inability to simulate CFD. Improving intervertebral soft tissue failure definitions will be essential to enhancing model reliability for HFI‐induced CFD, and will likely require direction‐ and mode‐dependent criteria (e.g., multi‐axial failure surfaces informed by combined‐loading experiments).

Despite modifications to the scalp and skull material definitions, excessive head rebound was observed in the simulations, which were not observed in the experiment. This discrepancy likely reflects a combination of scalp constitutive behavior during unloading, skull boundary stiffness, and plate‐scalp contact definitions. Postimpact unloading and hysteresis are commonly simplified in contemporary finite element head models [40, 41], yet they can strongly influence rebound and the early force time history in head‐first impact [42]. Although the skull and scalp updates, including the plate‐scalp friction definition, improved the suitability of the model setup for the loading mode studied, they were not subjected to a formal sensitivity analysis; therefore, absolute early‐phase load magnitudes should be interpreted with some uncertainty.

## 5 Conclusion

A detailed head–neck finite element model was enhanced to improve its suitability for inverted‐drop HFI simulation. Parametric simulations showed that increasing anterior head eccentricity while maintaining a horizontal Frankfort plane reduced cervical lordosis, promoted a more pronounced S‐shaped deformation during carriage loading, and increased C7/T1 anterior shear force and flexion moment, with a marked increase above 15 mm. A matched cadaveric inverted‐drop experiment at approximately 30 mm eccentricity produced bilateral C7/T1 CFD, consistent with the loading and deformation trends predicted by the simulations. Although complete dislocation was not reproduced in the model, comparison with the experiment indicated that current intervertebral soft‐tissue failure formulations limited simulation of the observed failure progression. Together, these findings suggest that preimpact head‐forward eccentricity can substantially increase lower‐neck loading and susceptibility to lower‐neck CFD under the inverted‐drop conditions studied.

## Author Contributions

**Mario Mongiardini:** methodology, investigation, formal analysis, writing – reviewing and editing, funding acquisition, visualization. **Duane Cronin:** methodology, writing – reviewing and editing. **Ryan D. Quarrington:** conceptualization, methodology, investigation, formal analysis, writing – original draft, writing – reviewing and editing, visualization, resources, supervision, funding acquisition, project administration.

## Funding

This research was supported by a Lifetime Support Authority project grant (R2230; R.Q., M.M.).

## Conflicts of Interest

The authors declare no conflicts of interest.

## Supporting information


**Data S1:** GHBMC head model modifications.
**Figure S1:** Measurement of scalp–impact plate friction. (A) Experimental setup used to estimate the kinetic coefficient of friction between human scalp skin and the drop tower aluminum impact plate. (B) Horizontal (tangential) force‐time traces for the three repeat sliding tests; shaded intervals indicate the steady‐sliding windows used to compute the mean tangential force for each trial.
**Figure S2:** Experimental and simulated vertical head impact load during the early phase of the drop test. The modified skull replicates the experimental initial peak load more closely than the original model and also reduces the gap to the second peak load created by the initial bouncing.

**Data S2:** jsp270219‐sup‐0002‐SuppMat2.docx.
**Figure S3:** (A) Vertical force at the impact plate, (B) axial force at the torso‐end (carriage), (C) C7/T1 shear force (anterior shear = positive), and (D) sagittal plane moment (flexion moment = positive) traces for each simulation.

**Data S3:** jsp270219‐sup‐0003‐SuppMat3.docx.
**Figure S4:** Intervertebral flexion rotation at C2/C3, C5/C6, and C7/T1 during the HFI condition for experiment (dashed) and simulation (solid) with eccentricity of 30 mm. Positive rotation indicates flexion. The shaded region highlights the interval after experimentally observed posterior ligament failure at C7/T1 (approximately 9 ms), where the experimental response exhibits rapid, supraphysiologic flexion.

## Data Availability

The data that support the findings of this study are available from the corresponding author upon reasonable request.
