# Supplementary material for: The Effect of Head‐Forward Posture on Lower Neck Loads and Dislocation Risk in Head‐First Impact: A Computational and Experimental Investigation
Source: JOR Spine. 2026 Sep 7;9(3):e70219. doi: 10.1002/jsp2.70219 (PMC13551008; doi:10.1002/jsp2.70219)
Supplement: Supplementary file 1 [file JSP2-9-e70219-s001.docx]

# SUPPLEMENTARY MATERIAL 1 - GHBMC head model modifications

**BACKGROUND AND MOTIVATION**

Preliminary results from a recent combined computational and experimental study of head-neck inverted drops highlighted some limitations of the GHBMC M50-HN response to HFI (Mongiardini et al., 2024). High peak impact forces and excessive head bouncing, relative to the experiments, occurred in the simulations and were consistent with observations from similar HFI simulations (Darweesh, 2022). These model limitations obscured detailed analysis of the head-neck mechanics immediately following impact, and may confound simulation-based investigations of HFI and CFD.

Discussions with the GHBMC developers revealed several issues with the head-neck model that may have contributed towards the unrealistic head impact response. Therefore, an in-house model validation pilot study was performed to improve the biofidelity of the GHBMC M50-HN response to HFI.

**METHODS**

***Inverted drop HFI simulation setup***

A previously validated modified version of GHBMC M50-HN head-neck model was used as the baseline model to simulate inverted drop HFI experiments (Darweesh, 2022). To replicate the conditions of prior HFI experiments (Nightingale et al., 1996), the neck flesh, all musculature, tendons, hyoid bone and mandible, and all attachments were removed from the original GHBMC head-neck model, and the model was rotated so that T1 was 25° relative to the horizontal plane. Model pre-positioning was performed to produce a head-neck posture with horizontal Frankfort Plane and 30 mm head eccentricity, ensuring that the model response was validated for the HFI with a pre-impact head-forward posture.

The inferior portion of the T1 vertebra was rigidly connected to a simplified geometrical representation of an instrumented overhead drop carriage (16 kg effective torso mass (Nightingale et al., 1996)) typically used in inverted drop test of head-neck assemblies. To simulate a typical drop rail, this simplified carriage assembly was constrained to vertical translation only. HFI was simulated by assigning an initial velocity of 2 m/s to the head-neck model, with the head positioned closely above the drop tower impact plate (modelled by a rigid surface). Loads acting on the impact surface and drop carriage were extracted from the simulations at 100 kHz.

***Scalp-plate coefficient of friction***

To maximise comparability between the simulations and experimental setup, friction tests were performed to estimate the kinetic coefficient of friction between human scalp skin and the aluminium impact plate of the drop tower, following the general principles of *ASTM G115* (Fig S-1; laboratory temperature maintained at 21 ± 1 °C). The head of a single fresh-frozen human cadaver (male, 70 years old; shaved) was rigidly mounted to a six-axis load cell (MC5-2500, AMTI, MA, USA). The aluminium impact plate was cleaned before each test (70% ethanol spray followed by cloth wipe), rigidly fixed to a horizontal linear bearing, and lowered until it rested on the scalp. The plate was vertically unconstrained and no additional vertical preload was applied; the normal force was therefore taken as the plate weight (7.33 N). The horizontal force was recorded while the plate was translated manually across the scalp via the bearing assembly (three repetitions). Sliding speed (42 ± 17 mm/s) was measured from planar video (100 Hz; Phantom VEO1010, Vision Research, USA). The coefficient of friction (μ) was calculated as the mean horizontal force during steady sliding divided by the normal force; initiation and termination transients were excluded. The mean μ across the three trials was 0.52, so this was assigned to the impact surface in the model.

*Fig S - 1: Measurement of scalp–impact plate friction. (A) Experimental setup used to estimate the kinetic coefficient of friction between human scalp skin and the drop tower aluminium impact plate. (B) Horizontal (tangential) force-time traces for the three repeat sliding tests; shaded intervals indicate the steady-sliding windows used to compute the mean tangential force for each trial.*

***Model modifications analysis***

Iterative modifications were made to the model’s scalp and the skull to better match the response to existing experimental HFI data (Fig S - 2). After each modification, an HFI simulation was performed and the impact surface and drop carriage load cell outputs were compared with corresponding HFI experimental data.

HFI was firstly simulated for the unmodified GHBMC M50-HN model to obtain baseline data for comparison. Next, the material properties of the GHBMC scalp flesh were replaced with those used in the Total Human Model for Safety (THUMS) (Iwamoto et al., 2002). In Model Iteration #3, erroneously duplicated inner and outer shell elements of the skull cortical bone were deleted, and the elastic modulus of the cortical bone solid elements was reduced from 15 GPa to 10 GPa.

Table S - 1: Iteration number for the modified head-neck models

| **Model #** | **Modifications** |
| --- | --- |
| 1 | Unmodified GHBMC M50-HN |
| 2 | GHBMC M50-HN with THUMS scalp material properties |
| 3 | Model 2 w/ shell elements deleted, cortical elements moduli set to 10 GPa |

**RESULTS**

Modifying the scalp material reduced head bouncing upon initial impact compared to the original GHBMC model (Fig S - 2). Although some residual bouncing remained, the modified head model re-established contact with the bottom plate sooner than the original, decreasing unloading of the partially loaded neck assembly and reducing the phase offset between the initial peak load and the subsequent major impact. Additionally, removing the inner and outer shell elements of the skull’s cortical bone, combined with lowering the elastic modulus of its solid elements, improved agreement between the simulated first peak load at the base of the neck (i.e., vertical force measured between T1 and the carriageway load cell) and experimental data. These modifications also reduced the time offset (*T_bounce_*) between the first simulated peak and the subsequent peak following head rebound.

Fig S - 2: Experimental and simulated vertical head impact load during the early phase of the drop test. The modified skull replicates the experimental initial peak load more closely than the original model and also reduces the gap to the second peak load created by the initial bouncing.

**REFERENCES**

Darweesh, M., 2022. Evaluating Cervical Spine Response and Potential for Injury During Head-First Impact Loading Using a Finite Element Head-Neck Model. University of Waterloo.

Iwamoto, M., Kisanuki, Y., Watanabe, I., Furusu, K., Miki, K., Hasegawa, J., Year Development of a finite element model of the total human model for safety (THUMS) and application to injury reconstruction. In Proceedings of the international IRCOBI Conference.

Mongiardini, M., Jones, C., Quarrington, R., Year The effect of head-forward posture on risk of lower neck dislocation during head-first impacts: a preliminary computational and dynamic experimental investigation. In Proceedings of the International Research Council on the Biomechanics of Injury. Stockholm, Sweden.

Nightingale, R.W., McElhaney, J.H., Richardson, W.J., Myers, B.S., 1996. Dynamic responses of the head and cervical spine to axial impact loading. J Biomech 29, 307-318.
