# Supplementary material for: The Effect of Head‐Forward Posture on Lower Neck Loads and Dislocation Risk in Head‐First Impact: A Computational and Experimental Investigation
Source: JOR Spine. 2026 Sep 7;9(3):e70219. doi: 10.1002/jsp2.70219 (PMC13551008; doi:10.1002/jsp2.70219)
Supplement: Supplementary file 2 [file JSP2-9-e70219-s002.docx]

# SUPPLEMENTARY MATERIAL 2

Figure S - 3: (A) Vertical force at the impact plate, (B) axial force at the torso-end (carriage), (C) C7/T1 shear force (anterior shear = positive), and (D) sagittal plane moment (flexion moment = positive) traces for each simulation.
