# Supplementary material for: The Effect of Head‐Forward Posture on Lower Neck Loads and Dislocation Risk in Head‐First Impact: A Computational and Experimental Investigation
Source: JOR Spine. 2026 Sep 7;9(3):e70219. doi: 10.1002/jsp2.70219 (PMC13551008; doi:10.1002/jsp2.70219)
Supplement: Supplementary file 3 [file JSP2-9-e70219-s003.docx]

# SUPPLEMENTARY MATERIAL 3

Fig S – Figure S – 4: Intervertebral flexion rotation at C2/C3, C5/C6, and C7/T1 during the HFI condition for experiment (dashed) and simulation (solid) with eccentricity of 30 mm. Positive rotation indicates flexion. The shaded region highlights the interval after experimentally observed posterior ligament failure at C7/T1 (approximately 9 ms), where the experimental response exhibits rapid, supraphysiologic flexion.
